# Parametric Design and Mechanical Characterization of a Selective Laser Sintering Additively Manufactured Biomimetic Ribbed Dome Inspired by the Chorion of Lepidopteran Eggs

**DOI:** 10.3390/biomimetics10010001

**Published:** 2024-12-24

**Authors:** Alexandros Efstathiadis, Ioanna Symeonidou, Emmanouil K. Tzimtzimis, Dimitrios Avtzis, Konstantinos Tsongas, Dimitrios Tzetzis

**Affiliations:** 1 Department of Architecture, University of Thessaly, 38221 Volos, Greece; 2 Digital Manufacturing and Materials Characterization Laboratory, School of Science and Technology, International Hellenic University, 57001 Thermi, Greece; 3 Forest Research Institute, Hellenic Agricultural Organization Demeter, 57006 Vassilika, Greece; 4 Department of Industrial Engineering and Management, School of Engineering, International Hellenic University, 57001 Thessaloniki, Greece

**Keywords:** biomimicry, ribbed domes, parametric design, 3D printing, SLS, mechanical behavior, finite element analysis, FEM

## Abstract

The current research aims to analyze the shape and structural features of the eggs of the lepidoptera species *Melitaea* sp. (Lepidoptera, Nympalidae) and develop design solutions through the implementation of a novel strategy of biomimetic design. Scanning electron microscopy (SEM) analysis of the chorion reveals a medial zone that forms an arachnoid grid resembling a ribbed dome with convex longitudinal ribs and concave transverse ring members. A parametric design algorithm was created with the aid of computer-aided design (CAD) software Rhinoceros 3D and Grasshopper3D in order to abstract and emulate the biological model. A series of physical models were manufactured with variations in geometric parameters like the number of ribs and rings, their thickness, and curvature. Selective laser sintering (SLS) technology and Polyamide12 (nylon) material were utilized for the prototyping process. Quasi-static compression testing was carried out in conjunction with finite element analysis (FEA) to investigate the deformation patterns and stress dispersion of the models. The biomimetic ribbed dome appears to significantly dampen the snap-through behavior that is observed in typical solid and lattice domes, decreasing dynamic stresses developed during the response and preventing catastrophic failure of the structure. Increasing the curvature of the ring segments further reduces the snap-through phenomenon and improves the overall strength. However, excessive curvature has a negative effect on the maximum sustained load. Increasing the number and thickness of the transverse rings and the number of the longitudinal ribs also increases the strength of the dome. However, excessive increase in the rib radius leads to more acute snap-through behavior and an earlier failure. The above results were validated using respective finite element analyses.

## 1. Introduction

Biomimicry is a relatively new interdisciplinary field of science that studies natural models and strategies, draws inspiration from them, and attempts to apply them to contemporary design and engineering challenges [1]. In order to facilitate the practice of biomimicry and bridge the knowledge gap between the different disciplines involved in the process [2], specialized methodological tools have been developed, like the Biomimicry Taxonomy [3,4,5]. Strategies of biomimetic design have also been proposed, like the top-down (or technology pull) and bottom-up (or biology push) methods developed by Knippers, both of which are characterized by a linear approach regarding the steps of the process [6,7,8]. The biomimetic design spiral is another strategy of biomimetic design developed by the Biomimicry Institute that attempts to tackle the challenge of biomimicry through a spiral and iterative process [4].

Algorithmic design is an iterative design process that involves a program or algorithm that meets certain user-defined parameters as input [9]. The output is an infinite set of possible design solutions [10]. The designer evaluates the output and has the ability to modify the input parameters or even the algorithmic source code in order to optimize the end result according to his design goals [11] and to data collected during the technical implementation process [12]. It has also enabled the generation of complex geometric models that would otherwise be extremely arduous to create [13]. Algorithmic design can find application in biomimetic design and in the strategies that were described earlier, particularly at the stages of abstraction and emulation of biological models.

Conventional manufacturing methods are unable to produce intricate biomimetic structures due to their technological limitations, which has also hampered the growth of biomimetic research and the development and application of biomimetic design solutions [14]. However, developments in additive manufacturing (AM) technologies have opened up new possibilities and made it possible to build complex structures and novel materials that were previously impossible [15,16]. More specifically, selective laser sintering (SLS) technology uses a laser beam to sinter a polymer powder and fuse the particles together, thus creating layer by layer the required product [17], a technology that has found application in the medical material technology and industrial sectors [18,19,20]. The advantages of the SLS technology include great design freedom, the complexity and high accuracy of printed parts, a variety of materials, minimal material waste, supportless printing, and good mechanical properties [21,22,23,24]. A disadvantage of the process, which is common to all laser-based AM technologies, is the high printing times [25].

An egg is an organic, hierarchical structure [26] with the function of carrying and incubating an embryo until its hatching [27]. Eggs are laid by various animals, ranging from insects and reptiles to birds, something that explains the great structural variety [28,29,30,31,32]. As eggs are essential not only for the nourishment, oxygen, and excretion exchange of the embryo [33,34] but also, more importantly, for protecting the embryo from unfavorable environmental conditions and threats, like crushing or puncturing [35,36], they have developed some excellent mechanical properties and features [37].

The goal of the present research is to study the structure of eggs of the nymphalid *Melitaea* sp. and to extract design solutions through the implementation of a novel strategy of biomimetic design. It also aims to incorporate algorithmic design tools in the abstraction phase of the biological model as it is transferred into a parametric digital model. Subsequently, the SLS additive manufacturing technology was selected in an attempt to integrate 3D printing technologies into the technical implementation stage of the biologically inspired design. Lastly, mechanical testing coupled with finite element analysis (FEA) was conducted to evaluate the technical properties of the biomimetic structure.

## 2. Materials and Methods

### 2.1. Novel Biomimetic Design Strategy

**Figure 1:**
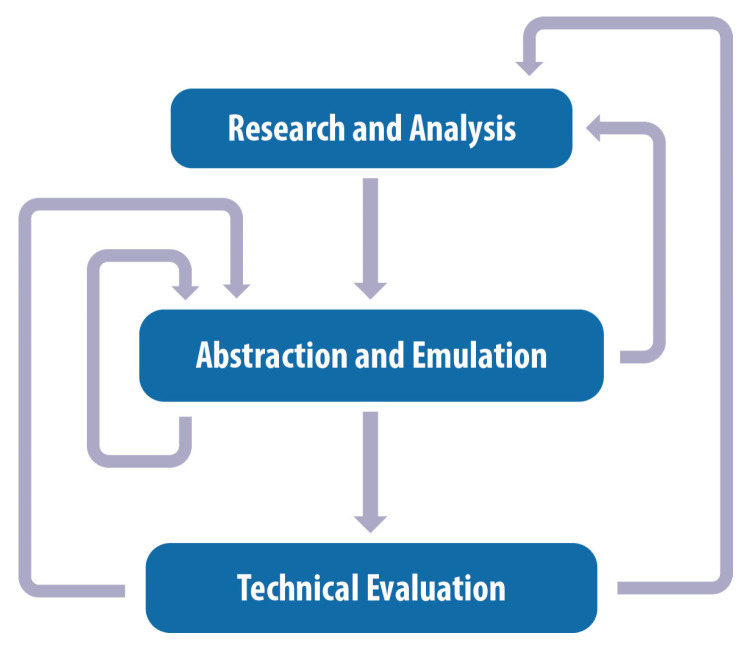
The novel biomimetic design strategy is characterized by by-directional feedback loops between its three stages: the “Research and Analysis” stage, followed by the “Abstraction and Emulation” stage, and concluded with the “Technical Evaluation” stage.

A novel biomimetic design strategy has been developed as part of the present research and has been documented in previously published work [38,39,40,41,42]. The first stage is the “Research and Analysis” of the biological model, which comprises biological research, the identification of biological structures of interest, and their subsequent analysis with means of microscopic analysis. This is followed by the “Abstraction and Emulation” stage, which consists of the extraction of the biological model and its transfer into a digital 3D model with the aid of computational design. The last stage is the “Technical Evaluation” of the biomimetic design concept, which involves creating prototypes with the aid of 3D printing technologies and assessing the mechanical behavior of the structure with appropriate testing and simulations. The three stages of the methodology are not linear, but instead, they are interconnected by bi-directional loops of feedback, as shown in Figure 1, where each phase informs the rest, and revisiting a previous stage is often necessary for optimization purposes. This kind of approach constitutes a significant step over existing strategies that are either linear or spiral [4,6,7,8].

### 2.2. Research and Analysis

More specifically, biological research was initially conducted with the aid of specialized biomimetic tools and databases like AskNature [3], along with traditional literature resources. It was determined that the chorion of lepidopteran eggs is a hierarchical structure that could potentially serve as a valuable source of biomimetic design inspiration [34,43,44]. *Melitaea* is a genus of brush-footed butterflies (Nymphalidae) that comprises several species with worldwide distribution [45]. In Greece, this genus is represented by eight species that occur diversely all over the country [46]. The egg masses of *Melitaea* sp. were collected in the suburban forest of Thessaloniki and were immediately brought to the laboratory of Forest Entomology (Forest Research Institute, Hellenic Agricultural Organization Demeter). After carefully removing the egg masses from the plant tissues that were attached, the eggs were lyophilized in 2 mL Eppendorf vials.

Analysis of the lepidopteran egg structure at the microscopic level was conducted using the digital microscope Dino-Lite Pro HR-AD7013MZT (Dino-Lite Europe, Almere, The Netherlands). Furthermore, the microstructure of the chorion was examined under the Phenom ProX Desktop Scanning Electron Microscope (SEM) (Thermo Fisher Scientific, Waltham, MA, USA). Chorion samples were coated with gold (Au) at the Quorum SC7620 Sputter Coater (Quorum Technologies, Laughton, UK) to prevent thermal damage caused by electrical charging and were mounted on double-sided carbon tape [47]. Further analysis of the microscopic imaging was conducted on the ImageJ (v.1.53t, National Institutes of Health, Bethesda, MD, USA) software.

### 2.3. Abstraction and Emulation

Computer-aided design (CAD) software Rhinoceros 3D (v.Rhino 7, 7.1.20343.09491, Robert McNeel & Associates, Seattle, WA, USA) and the embedded visual programming environment Grasshopper 3D (v.1.0.0007, Robert McNeel & Associates, Seattle, WA, USA) were utilized for the abstraction of the biological model and its translation into a digital, three-dimensional model. Additionally, the volumetric modeling plug-in Denrdo (v1.0.0, ECR Labs, Los Angeles, CA, USA) was utilized, which has the advantage of generating light volume data structures that are faster to compute [48]. A series of models were generated with variable geometric parameters.

### 2.4. Technical Evaluation

Selective laser sintering (SLS) technology was selected for the fabrication of physical specimens of the biomimetic models. The Sinterit Lisa rev.A SLS printer (*Sinterit* sp. z o.o., Krakow, Poland) was used in combination with commercial Polyamide 12 (nylon) powder (*Sinterit* sp. z o.o., Krakow, Poland). An advantage of the SLS technology is that there is no need to add support, as the powder itself performs this role. Avoiding the use of supports was considered necessary due to the geometric intricacies of the structure. The nesting and slicing of the models were carried out on the Sinterit Studio slicer (v.1.8.1.1, *Sinterit* sp. z o.o., Krakow, Poland). The specimens were carefully extracted from the powder “cake” and cleaned in order to avoid damaging the overall structure. For the same reason, no sandblasting was employed in the process.

Three specimens of each model were tested for reliability purposes under uniaxial quasi-static concentrated compression loading at room temperature at a universal testing machine (M500-50AT Testometric Company, Rochdale, UK) equipped with a 50 kn load cell and a strain rate of 5 mm/min in order to identify trends in the mechanical performance of the biomimetic structure in relation to changes in its topological characteristics. The mechanical characteristics of the biomimetic ribbed domes were explored using the ANSYS™ software (ANSYS, Inc., Canonsburg, PA, USA). To precisely replicate the mechanical response of the domes, an explicit dynamic analysis was executed, as it was vital for representing their extensive distortions and bi-linear material behavior. To confirm results unaffected by mesh density, a convergence assessment was executed. This analysis indicated that stress convergence was attained with roughly 250,000 nodes and 670,000 elements for each validation model.

## 3. Biomimetic Design Process

### 3.1. Morphological Analysis of the Biological Sample

**Figure 2:**
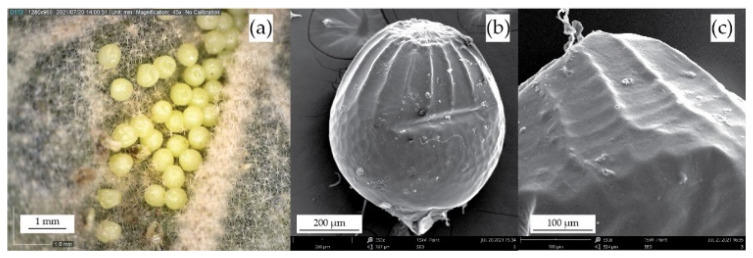
Imaging of the eggs of *Melitaea* sp.: (**a**) the eggs under optical microscopy as seen deposited on a leaf; (**b**) a single egg under SEM with its three zones—apical, medial, and basal—visible; (**c**) close-up of the medial zone where the longitudinal ribs and transversal rings form an arachnoid grip pattern.

The eggs of *Melitaea* sp. have the shape of slight prolate spheres with a diameter ranging from 0.55 mm to 0.63 mm, as illustrated in Figure 2a,b and documented in relevant literature [43]. SEM analysis of the eggs reveals that the outer layer of the egg, known as the chorion, is a hierarchical structure [49], as seen in Figure 2b. It is characterized by three distinct areas: the apical zone, consisting of elongated hexagons (also known as the rosette); a medial zone, consisting of curved longitudinal ribs and transverse rings; and a basal zone, formed by irregular hexagons [38]. The convex ribs, along with the concave rings, form an arachnoid micro-grid pattern resembling a ribbed dome [34,50] that can be distinguished in Figure 2c. The role of this structure is to enhance the structural stability of the eggshell [27,35]. The rib count was calculated at 16–18 and the ring count at 12–14, while the ribs were more pronounced than the rings with a relative thickness ratio of 1.51–1.62. This arachnoid pattern is what distinguishes the specific biological structure and served as a source of inspiration for the design of a biomimetic ribbed domed.

### 3.2. Geometric Characterization of a Ribbed Dome

**Figure 3:**
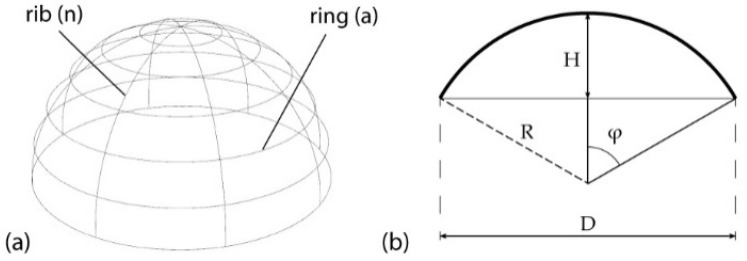
Main geometric characteristics of a dome: (**a**) longitudinal ribs (n) and transverse rings (a); (**b**) height (H), its span (D), and the total subtended angle (φ).

An advantage of dome structures is that they can cover the largest volume of space with the smallest use of material, resulting in constructions of minimal weight, while the nonaxial orientation of their elements provides excellent deformation performance to the overall structure through even propagation of stresses [51,52]. A ribbed dome generally comprises ribs and rings, where ribs are components that are distributed in the longitudinal direction while rings are components laid in the transverse direction [53]. Thus, typical ribbed domes are characterized by the following geometric parameters, as illustrated in Figure 3 [52]:

- The number of ribs (n) and number of rings (a).
- The height (H) and the span (D) of the dome.

Other parameters that define a dome is the total subtended angle, which is calculated from its height-to-span ratio, more specifically from the following equation [54]:

(1) $$\tan\varphi =\frac{4\times \frac{D}{H}}{{\left(\frac{D}{H}\right)}^{2}-4}\;,$$

**Figure 4:**
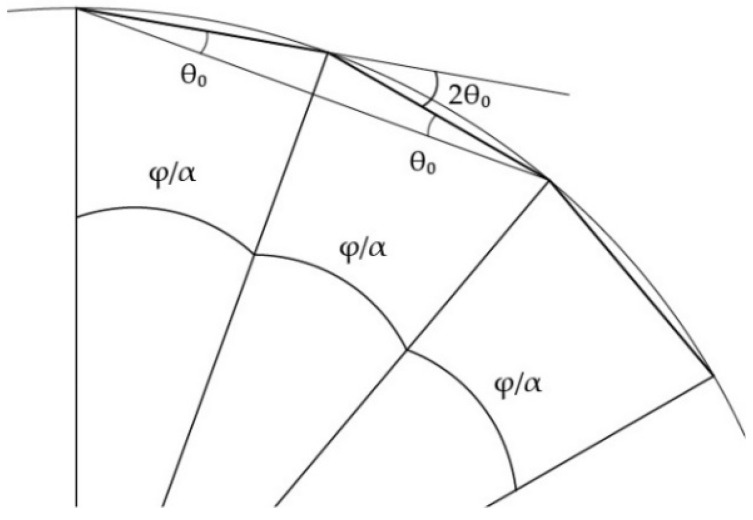
Longitudinal cross-section of a ribbed dome with ring member angle φ/α and rib member angle 2θ_0_.

Lastly, the angle that is created between rib members separated by the rings, as illustrated in Figure 4 is equal to 2θ_0_ [55]

(2) $$\theta_{0}=\frac{\varphi }{2\alpha }\;$$

where α is the number of rings.

### 3.3. Algorithmic Design

**Figure 5:**
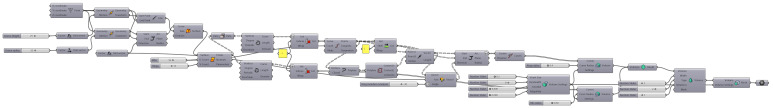
The interactive algorithm in the Grasshopper environment.

**Figure 6:**
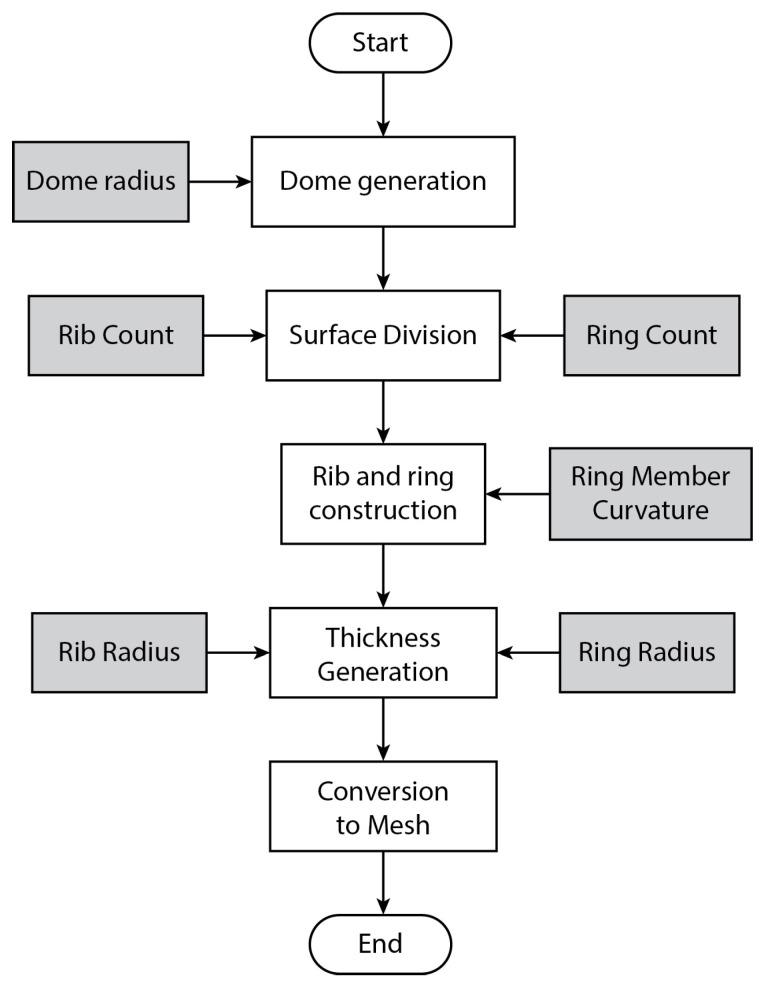
Top-level workflow diagram of the design algorithm of the biomimetic ribbed dome. Important parameters are seen in light gray boxes.

A parametric algorithm was created with the aid of Grasshopper visual programming language, which produces a digital model mimicking the chorion’s medial zone structure. Figure 5 shows the complete definition with all its components as found on the Grasshopper canvas. At the top algorithmic level, illustrated in Figure 6, a dome is initially created with a user-defined radius. The next step is to subdivide the surface of the dome, subsequently generating longitudinal ribs and transverse rings with variable counts. The curvature of the individual concave ring members can also be customized. In the end, thickness is given to the ribs and rings, and a final mesh is created.

**Figure 7:**
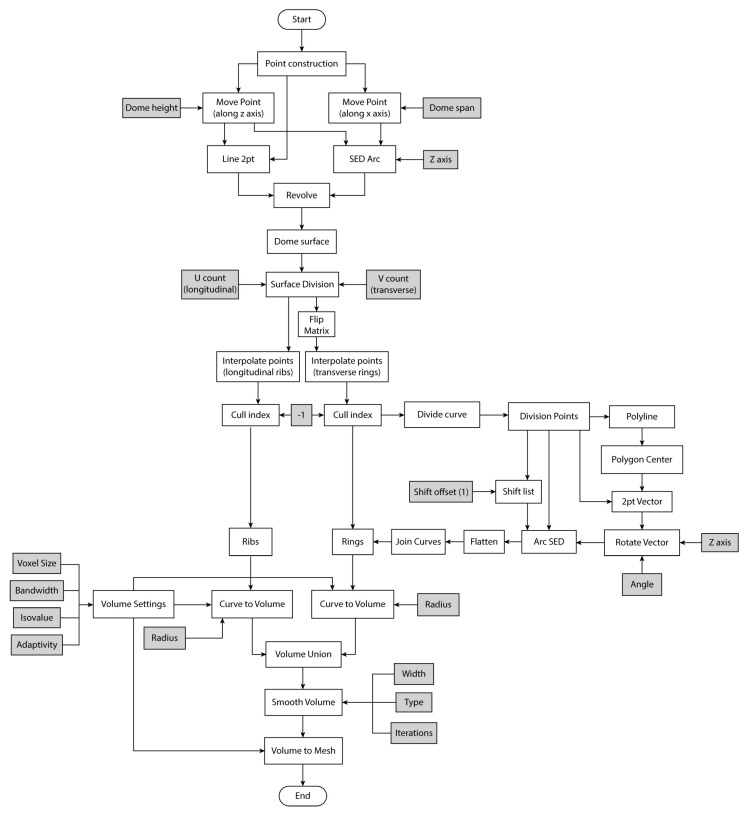
Second-level workflow diagram of the algorithmic generation of the biomimetic dome. Dependent parameters are in white and independent ones in light gray boxes.

A detailed, lower-level workflow diagram of the design algorithm can be found in Figure 7. Two points are initially constructed along the x axis and along the z axis of a cartesian three-dimensional space. These two points then form the start and end points, respectively, of an arc with a tangent at the start point, coincident with the z direction. The arc is then revolved, and a dome geometry is formed. The revolution axis is a line with the origin point as the start point and the z axis as the direction.

Next, the surface of the dome is divided into segments. The U and V divisions are customizable and determine the number of the final ribs and rings, respectively. The output points are then interpolated to form longitudinal curves. At the same time, their data matrix is flipped and interpolated to construct transverse curves from the same point set. The interpolation process creates a repeated curve that is culled.

Subsequently, divisions along the transverse curves are created, equal in number to the longitudinal curves. Closed polylines are constructed from the division points, and the centers of these polygons are extracted. Afterward, SED (Start, End, Direction) arcs are formed with the division points as the start points and the division points list offset by a count of 1 as the endpoints. The curvature of each arc is determined by the direction of a tangent vector that has the same starting point as the arc, the polygon’s center as an ending point, and can be rotated around the z axis to change the curve of the arc. In the end, the resulting curves are flattened and joined.

In the final step of the biomimetic dome generation process, thickness is added to the longitudinal ribs and transverse rings with the application of specific components from the Dendro plug-in, like “Curve to Volume”. Dendro Volumes are created for the two sets of curves with a user-defined radius for each of the sets. A volume union operation is performed coupled with a smoothness process. The final volume, a dendro specific geometry format, is converted into a mesh that can be further processed in Grasshopper or Rhino.

## 4. Results and Discussion

### 4.1. Algorithmic Design and SLS Printing Results

The result of the above algorithm is a parametric ribbed dome model with a series of user-defined design parameters. Parameters that produce significant changes in the structure’s geometry are the number of ribs and rings, their respective thicknesses, and the curvature of the ring members. A baseline model (Model 1) was generated with design parameter values analogous to the geometrical properties of the original biological structure in order to obtain a model qualitatively similar to the one shown in Figure 2b,c. The baseline biomimetic ribbed dome has a height of 25 mm, a span of 50 mm, and a 90° angle φ. It is also characterized by a rib count of 16, a ring count of 12, a rib and ring radius of 0.4 and 0.6, respectively, and a ring member curvature angle of −30° (angle of the tangent vector of the SED arc). The voxel size, bandwidth, isovalue, and adaptivity parameters were set as 0.2, 8, 0, and 0, respectively. Finally, the values of smooth width, type, and iterations are 1, mean, and 1, respectively. The complete set of design parameters of the baseline model are listed in Table 1.

**Figure 8:**
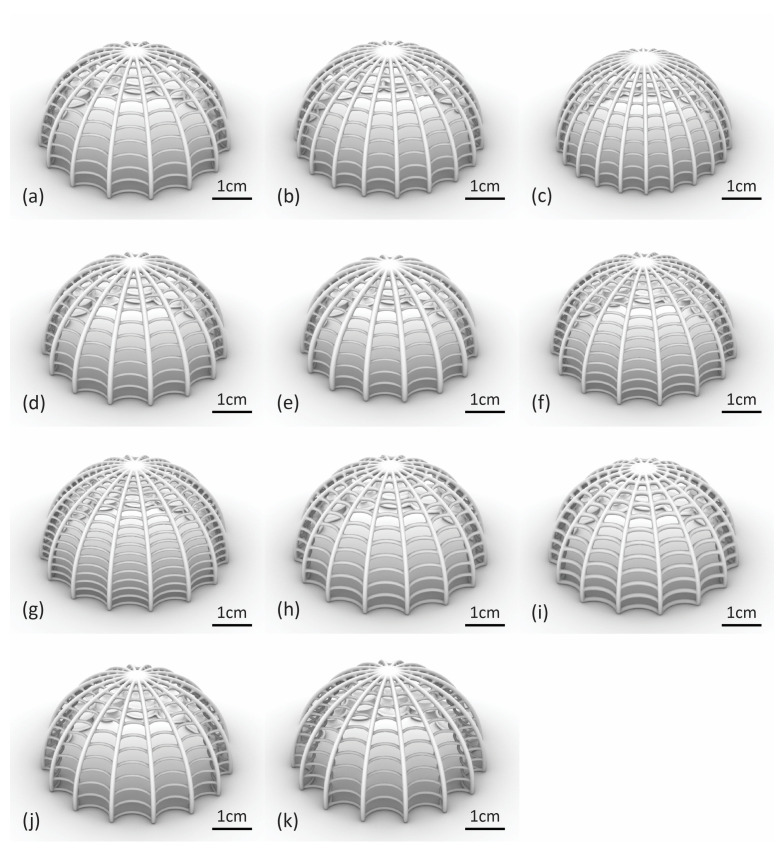
Digital models of the biomimetic dome structure: (**a**) Model 1 (baseline); (**b**) Model 2; (**c**) Model 3; (**d**) Model 4; (**e**) Model 5; (**f**) Model 6; (**g**) Model 7; (**h**) Model 8; (**i**) Model 9; (**j**) Model 10; (**k**) Model 11.

Variations in the baseline model were created with progressive alterations of its geometric characteristics. For Model 2, the rib count was changed to 20, and for Model 3 to 24. The rib radius in Model 4 was raised to 0.7 mm, and the rib radius of Model 5 to 0.8 mm. Similarly, the ring count of Model 6 was increased to 15 and for Model 7 to 18. The ring radius was raised to 0.5 mm and 0.6 mm for Models 8 and 9, respectively. Lastly, the ring member curvature angle was changed to −15° for Model 10 and to 0° for Model 11. The complete sets of altered parameters can be found in Table 2, and all the corresponding digital models in Figure 8.

**Figure 9:**
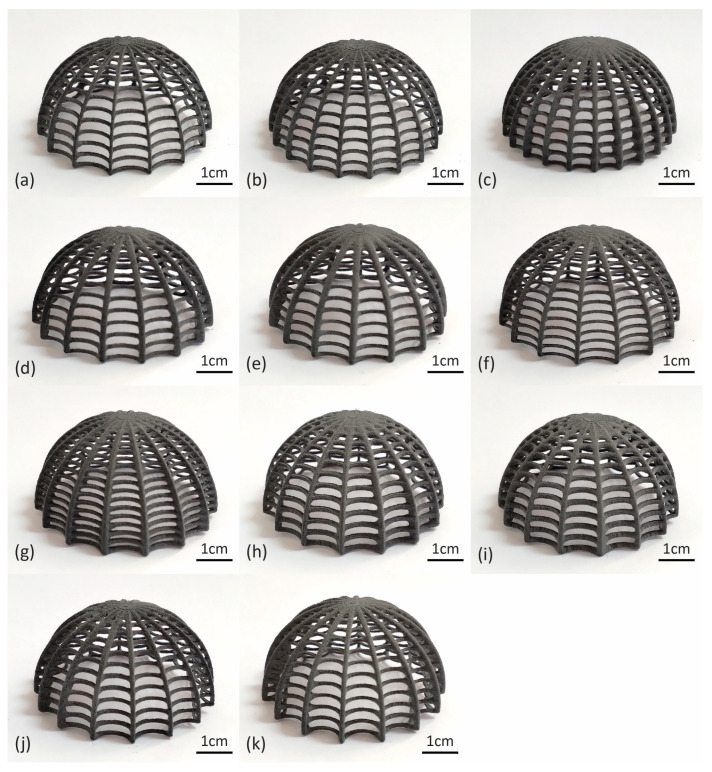
Three-dimensionally printed specimens of the biomimetic dome structure: (**a**) Model 1 (baseline); (**b**) Model 2; (**c**) Model 3; (**d**) Model 4; (**e**) Model 5; (**f**) Model 6; (**g**) Model 7; (**h**) Model 8; (**i**) Model 9; (**j**) Model 10; (**k**) Model 11.

Physical samples of each design variation were printed on the SLS platform and can be seen in Figure 9. As for the printing parameters, a layer height of 0.125 mm was selected. The laser power ratio was maintained at 1.00, and the Print Surface Temperature Offset at 0 °C. The shrink ratio was also left unchanged at 1.000 × 1.000 × 1.000. In each print, 8–9 specimens were placed simultaneously to save time, resulting in a total of four prints for the 33 total physical specimens requested. The total print time was, on average, 13 h and 50 min. The required volume of the polyamide powder (power needed in the feed bed) was estimated at 3.82 L on average, and the volume of the refresh powder after print was estimated at 0.86 L (avg). The total feed bed powder height was estimated at 9.6 cm (avg). All the printer parameters can be found in Table 3.

### 4.2. Characterization of the Compressive Behavior of the 3D-Printed Biomimetic Ribbed Dome Structure

**Figure 10:**
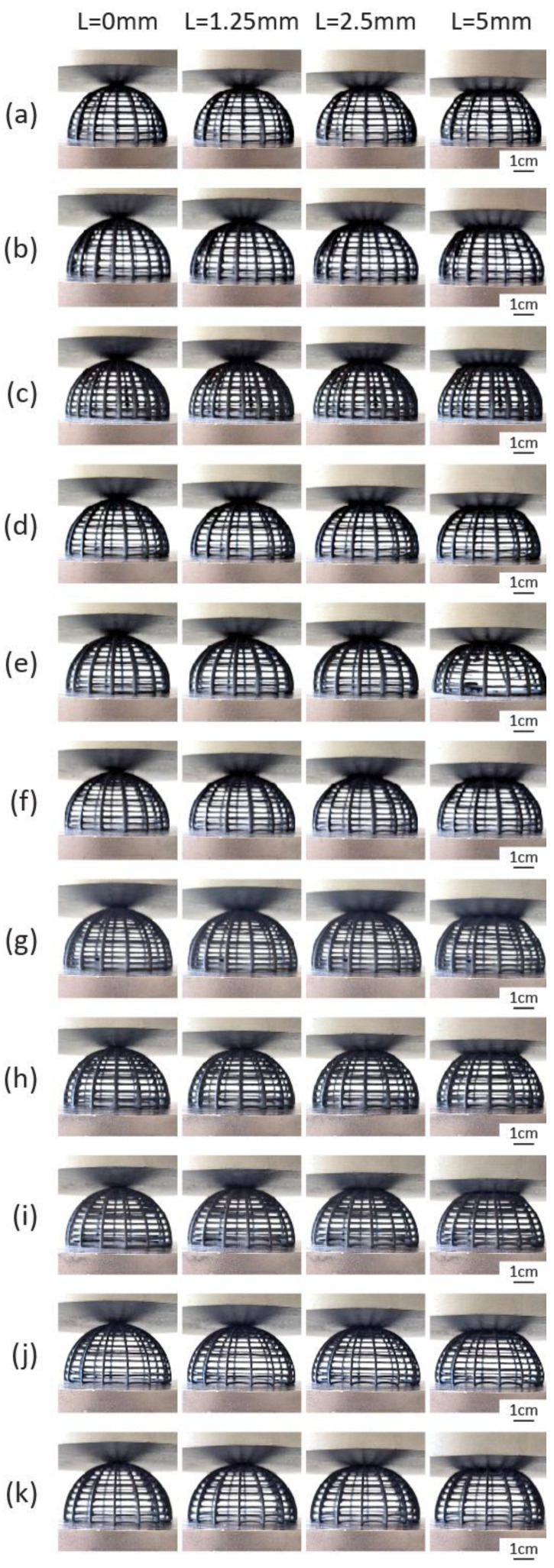
Compressive behavior at 0 mm, 1.25 mm, 2.5 mm, and 5 mm of (**a**) Model 1 (baseline); (**b**) Model 2; (**c**) Model 3; (**d**) Model 4; (**e**) Model 5; (**f**) Model 6; (**g**) Model 7; (**h**) Model 8; (**i**) Model 9; (**j**) Model 10 and (**k**) Model 11.

**Figure 11:**
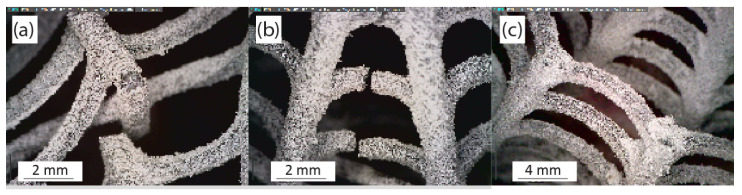
Failure points of the ribbed dome: (**a**) buckling and fracture of individual rib members; (**b**) buckling and fracture of individual ring members; (**c**) line instability, where a whole ring collapses.

Compression testing of the biomimetic ribbed dome structure reveals a minor snap-through (buckling) behavior that is typical in both solid and lattice domes [56,57]. During this response, a sudden transition occurs in the structure of the dome when a critical load is reached, which can be attributed to node (the joining point of members) instability as certain nodes sustain larger deflections than adjacent nodes due to higher localized stress [52,58,59]. As a result, the nodes collapse to a secondary equilibrium state upon reaching a critical displacement, which is typically characterized by a reverse curvature [60,61]. This behavior can be observed in the second column of the compression frames found in Figure 10, where the onset of the snap-through event occurs at the top of the dome, where the nodes snap and collapse below the compression level. As the event is not very pronounced, it is slightly obscured by the loading element. Subsequently, the structure continues to buckle until it reaches peak load, and the ultimate failure of the dome ensues, as seen in the final frames of Figure 10. Other failure mechanisms involve buckling and fracturing of individual rib or ring members, as seen in Figure 11a,b or total collapse of nodes along a ring, as seen in Figure 11c [52,59,62].

**Figure 12:**
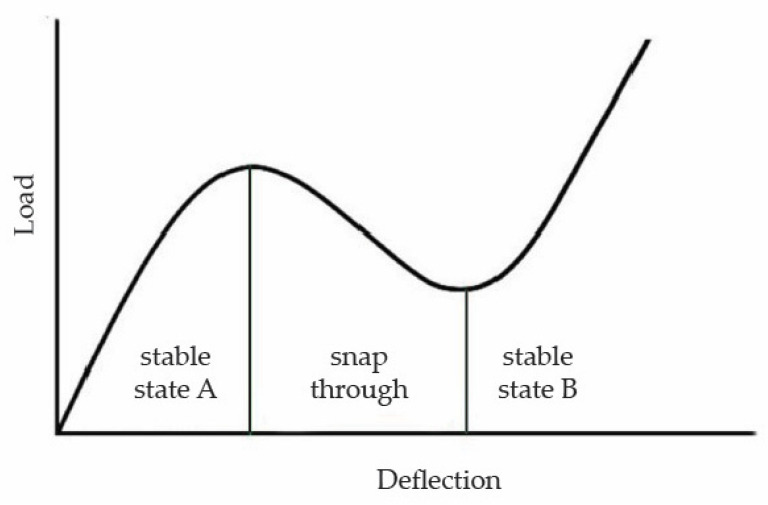
A typical curve of the snap-through response is observed in a solid or regular ribbed dome under compressive load.

**Figure 13:**
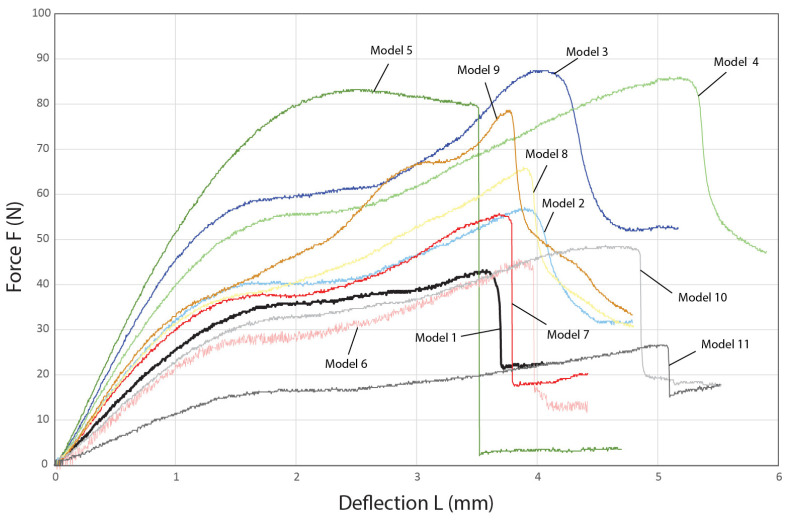
Load–deflection curves of the 3D-printed biomimetic domes when tested under compressive load.

A typical snap-through response curve of solid or lattice domes is illustrated in Figure 12, which is characterized by stark changes in the curve’s slope as the structure moves from stable state A to stable state B, indicating an intense snap-through phenomenon [63,64]. Furthermore, the respective load-deflection curves of the biomimetic ribbed domed specimens can be found in Figure 13. It becomes obvious that the snap-through is significantly dampened in all the tested specimens when compared to the typical curve in Figure 12. Avoiding an intense response of this kind is desirable as the dynamic loads that are developed in the process can prove detrimental to the overall structure [52]. The catastrophic collapse of Model 5 during the snap-through could be regarded as the result of such a mechanism. Thus, raising the rib radius above 0.7 mm would not improve the mechanical performance of the dome as the ensuing aggressive loads during the snap-through phase would lead to the abrupt failure of the dome. The dampening effect of this mechanism observed in the biomimetic structure can be attributed to the biomimetic arachnoid structure and, more specifically, to the concave transverse ring members, which distinguishes the model from a standard lattice dome, as increasing their curvature appears to further reduce the effect as seen in the curves of Models 10 and 11. The design solution abstracted from the eggs of the nymphalid *Melitaea* sp. efficiently tackles this particular behavior of a ribbed dome when it sustains concentrated compressive loads that could otherwise be disastrous to its structural integrity.

The maximum sustained load of the baseline model (Model 1) is 42.1 ± 2.44 N, as seen in Table 4. When the longitudinal rib count is raised to 20 in Model 2 and 24 in Model 3, the developed max sustained loads increase to 58.33 ± 1.5 N and 87.77 ± 2.27 N, respectively. Increasing the rib radius to 0.7 mm in Model 4 also increases the max load to 87.2 ± 2.27 N. However, when the radius is set to 0.8 mm in Model 5, the highest calculated load drops to 83.03 ± 5.05. As mentioned earlier, increasing the rib radius after a certain point apparently exacerbates the snap-through response, thus creating dynamic loads leading to an early structural failure. Model 6, which is characterized by a ring count of 15, sustains 45.27 ± 2.32 N of maximum compressive load, an increase compared to the baseline. The max load is further enhanced to 51.47 ± 6.97 N when the transverse rings are set to 18 in Model 7. A similar trend is observed when progressively changing the thickness of the rings to 0.5 mm in Model 8 and to 0.6 mm in Model 9. The highest developed loads are determined to be 66.47 ± 6.31 and 76.87 ± 11.1 N, respectively. Finally, increasing the curvature of the ring members, more specifically changing the angle of the tangent vector of the SED arc to −15°, raises the max load to 47.1 ± 2.19 N. However, further altering the angle to 0° decreases the calculated max load to 26.6 ± 1.4 N, which is significantly lower than even the one observed in Model 1. It becomes apparent that even though the biomimetic design solution of the concave ring members effectively deals with the snap-through behavior, there is a certain limitation to increasing the curvature without compromising the overall strength of the dome.

### 4.3. FEA Validation of Experimental Results

A confirmed material model derived from a numerical-experimental nanoindentation method [65] within the finite element analysis (FEA) was incorporated into the FE model to evaluate how the biomimetic structures perform under compression. Additionally, a computational model was employed to assess how 3D-printed structures respond to compression-induced stress. The biomimetic structures underwent incremental vertical velocities applied to the top plate, while the corresponding reaction forces were recorded at the simple support bottom boundary. The real vertical displacement values were determined from experimental outcomes and imported into the FE model.

**Figure 14:**
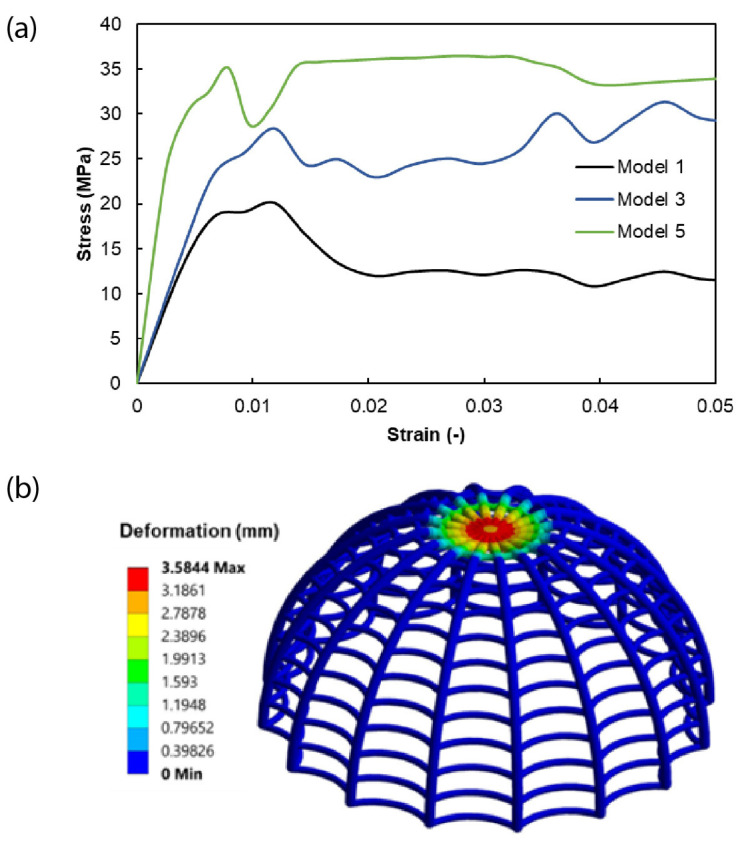

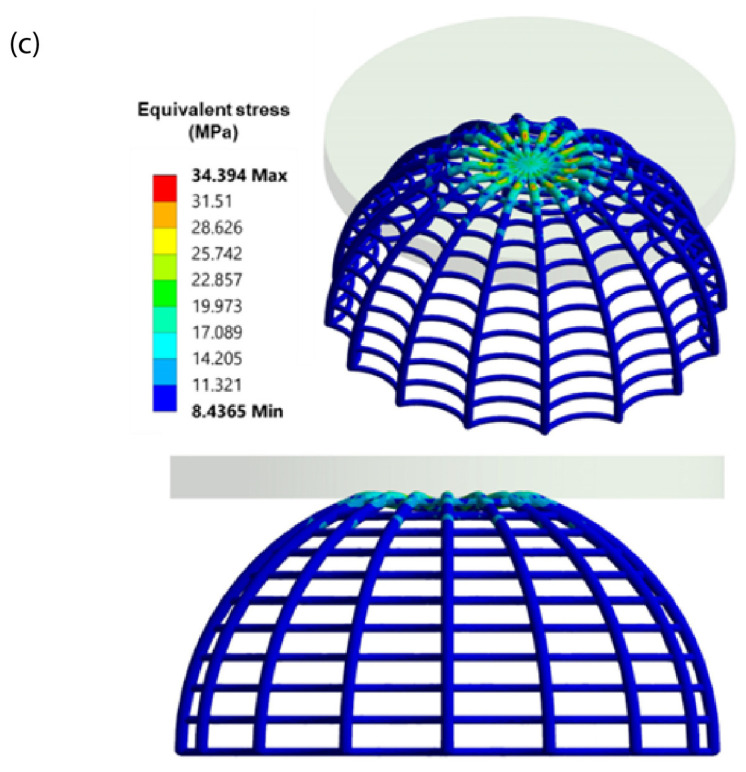
(**a**) The stress–strain behavior of the biomimetic lattice structures generated by finite element analysis (FEA), (**b**) vertical deformation, and (**c**) stress distribution of the biomimetic structure under compression load were analyzed using the material properties of PA12 within the FE model.

Tetrahedral elements were used for the ribs of the domes, while hexahedral elements were employed for meshing the upper compression plate. Figure 14a illustrates the stress–strain behavior acquired through finite element analysis (FEA) for the baseline and the stiffer model, i.e., Model 5. Model 1 serves as the baseline with a certain stress level, and in Model 3, which is characterized by a higher longitudinal rib count, the stiffness and the stress further increase. In contrast, Model 5, featuring a specific rib radius, experiences a notable change in stress and stiffness. This phenomenon emphasizes the influence of rib radius adjustments on structural performance. Consequently, the outcomes displayed in Figure 14b,c, depicting deformation and equivalent stress distribution for the 3D-printed biomimetic structures under compressive load, accurately pinpoint regions with high stress within the structures.

In contrast to prior investigations [65,66,67,68,69,70,71,72,73], lattice structures similar to our present study showcased enhanced physical and mechanical attributes, particularly in terms of significantly improved compressive strength and energy absorption. Conclusions drawn from mechanical test results indicate that combining computationally generated (FEA) compression test data with actual measurements could be an efficient approach for characterizing the mechanical deformation behavior of 3D printed biomimetic configurations.

## 5. Conclusions

Microscopic analysis of the shell of the eggs of lepidoptera *Melitaea* sp. ova reveals a ribbed dome which in its middle zone consists of longitudinal ridges and transverse rings composed of concave segments. The transfer of the above structure into a digital model was performed using the software Rhinoceros 3D (v.Rhino 7, 7.1.20343.09491) and Grasshopper 3D (v.1.0.0007). A parametric algorithm was designed, analogous to the biological structure, which allows the adjustment of a number of geometric parameters, such as the number and thickness of the rings and rings, as well as the curvature of the concave transverse segments. A total of 11 design variations were created and physical specimens of the models were fabricated using SLS 3D printing technology and polyamide 12 (nylon) powder.

The physical specimens of the biomimetic ribbed dome were subjected to technical compression tests, which reveal a significant attenuation of the snap-through effect that generally characterizes dome behavior under compressive load. The avoidance of this behavior is desirable as it is accompanied by intense dynamic loads that can lead to catastrophic collapse and can be attributed to the biomimetic design solution of the concave transverse ring sections. Enhancing the curvature of the ring segments significantly reduces the snap-through phenomenon and improves the strength of the structure up to a certain point since considerable weakening of the dome is observed when the curvature is increased more than 0° of the angle of the tangent vector of the SED arc. The maximum load also increases as the number and thickness of the transverse rings and the number of the longitudinal ribs increase. However, raising the radius of the ribs has a positive effect on the overall strength only up to a certain point, as when it reaches a value of 0.8 mm, the snap-through response is aggravated, resulting in premature failure of the structure. Clearly, there is a trade-off between the overall compressive load resistance of the structure and the intensity of the snap-through behavior, expressed geometrically through the above parameters. Moreover, a computational model was utilized to evaluate the response of 3D printed structures to stress induced by compression. The lattice structures demonstrated heightened physical and mechanical characteristics, notably showing substantial enhancements in both compressive strength and energy absorption. This approach is deemed efficient in characterizing the mechanical response of such structures. Future research could focus on further developing the case studies conducted in this research with a view to applying them to specific design issues. The design solution of ribbed domes with concave ring sections, could find application in architectural-scale vaulted structures and in vaulted vessels where it is important to avoid sudden loss of structural integrity.

## Figures and Tables

**Table 1:** The complete design parameters of the baseline (Model 1) biomimetic ribbed dome.

Design Parameter	Value
Dome Span	50 mm
Dome Height	25 mm
U count	16
V count	12
SED angle	−30°
Curve Radius (ribs)	0.6 mm
Curve Radius (rings)	0.4 mm
Voxel Size	0.2
Bandwidth	8
Isovalue	0
Adaptivity	0
Smooth Width	1
Type	2 (mean)
Iterations	1

**Table 2:** Geometric parameters of all biomimetic model permutations.

Model	Rib Count	Rib Radius (mm)	Ring Count	Ring Radius (mm)	Ring Curvature
1	16	0.6	12	0.4	−30°
2	20	0.6	12	0.4	−30°
3	24	0.6	12	0.4	−30°
4	16	0.7	12	0.4	−30°
5	16	0.8	12	0.4	−30°
6	16	0.6	15	0.4	−30°
7	16	0.6	18	0.4	−30°
8	16	0.6	12	0.5	−30°
9	16	0.6	12	0.6	−30°
10	16	0.6	12	0.4	−15°
11	16	0.6	12	0.4	0°

**Table 3:** SLS printer parameters that were used during the printing process of the biomimetic structures.

Printer Parameter	Value
Printer Model	Lisa rev.A
Material	Polyamide12 Smooth
Layer Height	0.125 mm
Laser Power Ratio	1.00
Print Surface Temperature Offset	0 °C
Shrink Ratio	1.000 × 1.000 × 1.000
Total Print Time (avg)	13 h 50 min
Powder Needed in Feed Bed (avg)	3.82 L
Refresh Powder After Print (avg)	0.86 L
Feed Bed Power Height (avg)	9.6 cm

**Table 4:** Maximum sustained loads of the 3D-printed models according to experimental testing.

Model	Load_max_ (N)
1	42.1 ± 2.44
2	58.33 ± 1.5
3	87.77 ± 2.27
4	87.2 ± 2.34
5	83.03 ± 5.05
6	45.27 ± 2.32
7	51.47 ± 6.97
8	66.47 ± 6.31
9	76.87 ± 11.1
10	47.1 ± 2.19
11	26.6 ± 1.4

## Data Availability

The raw data supporting the conclusions of this article will be made available by the authors on request.
